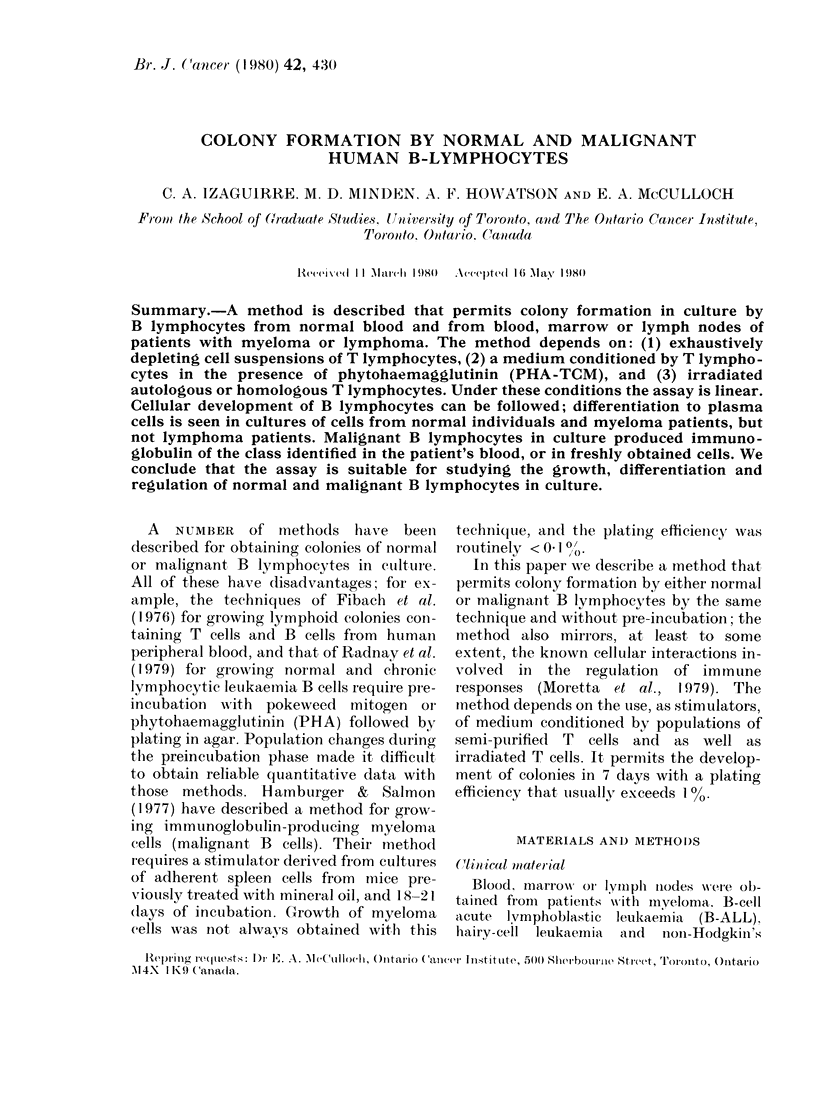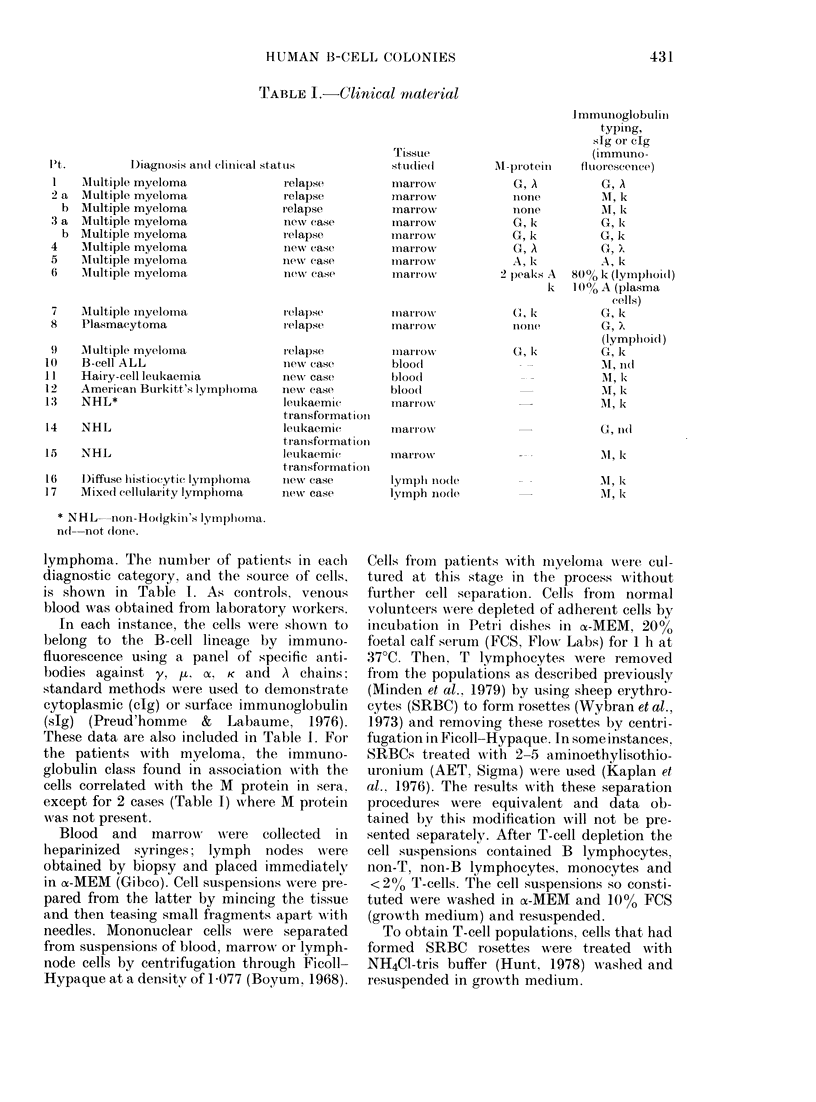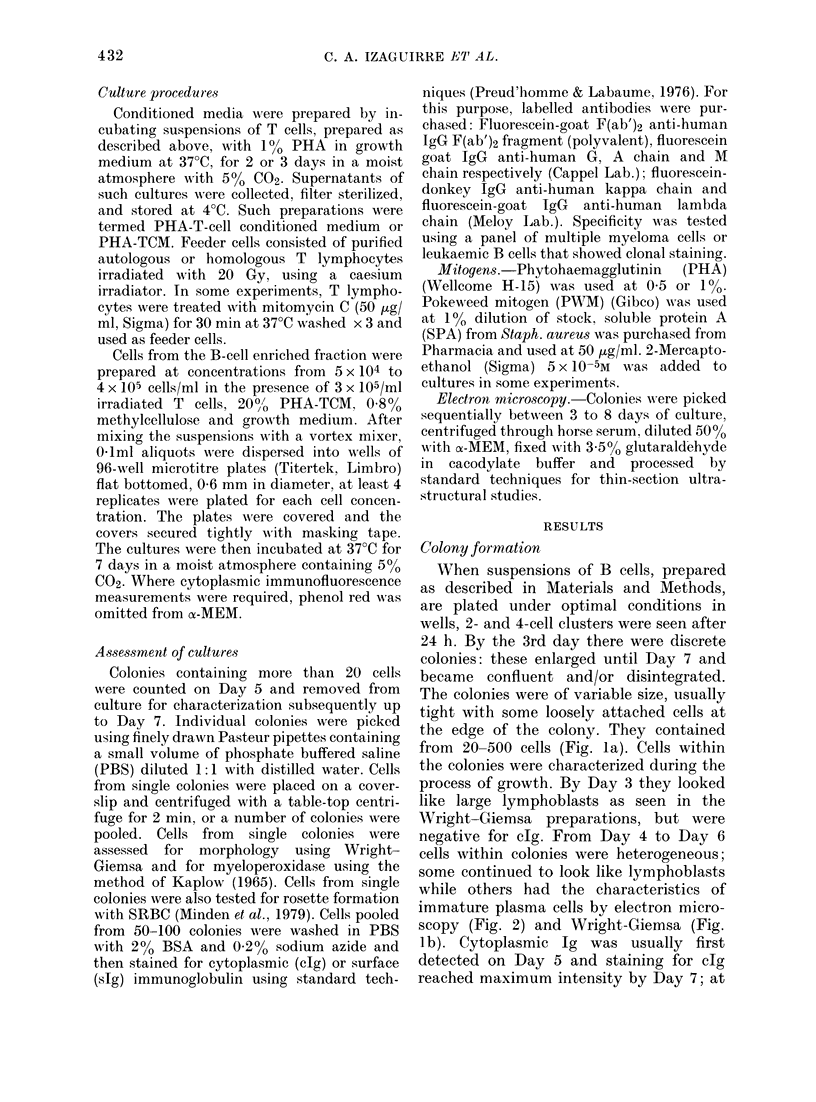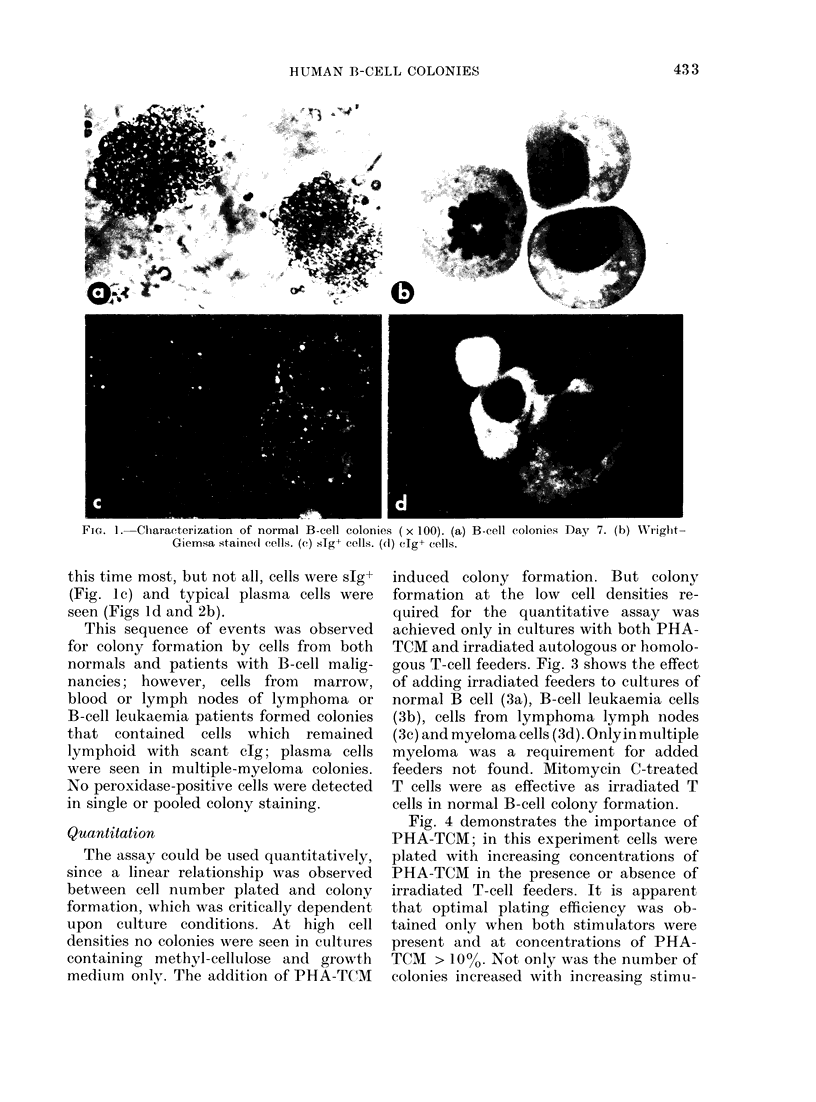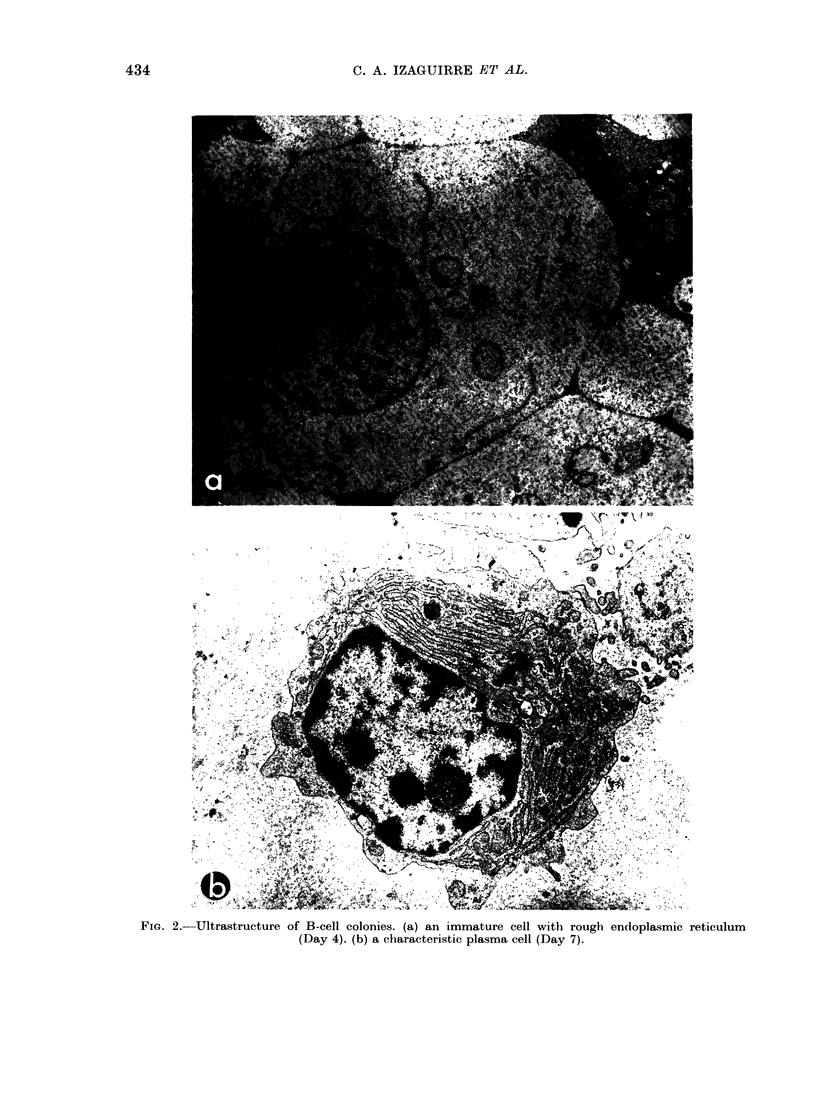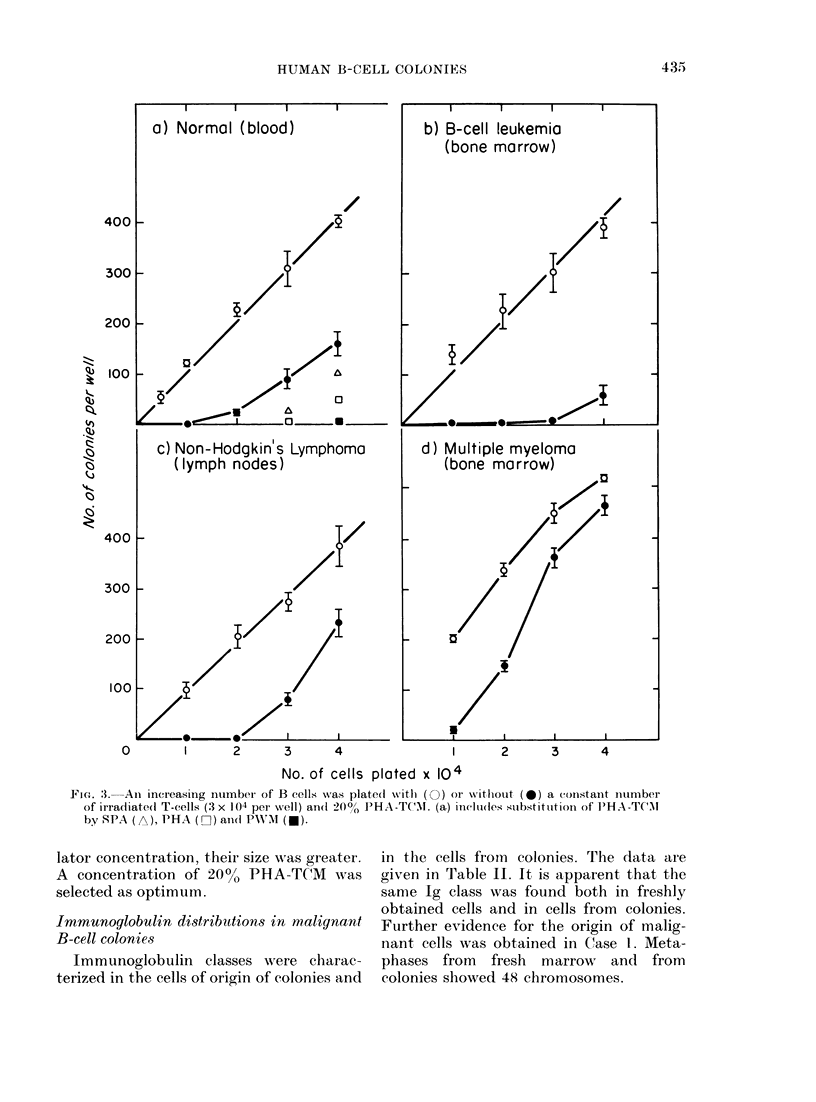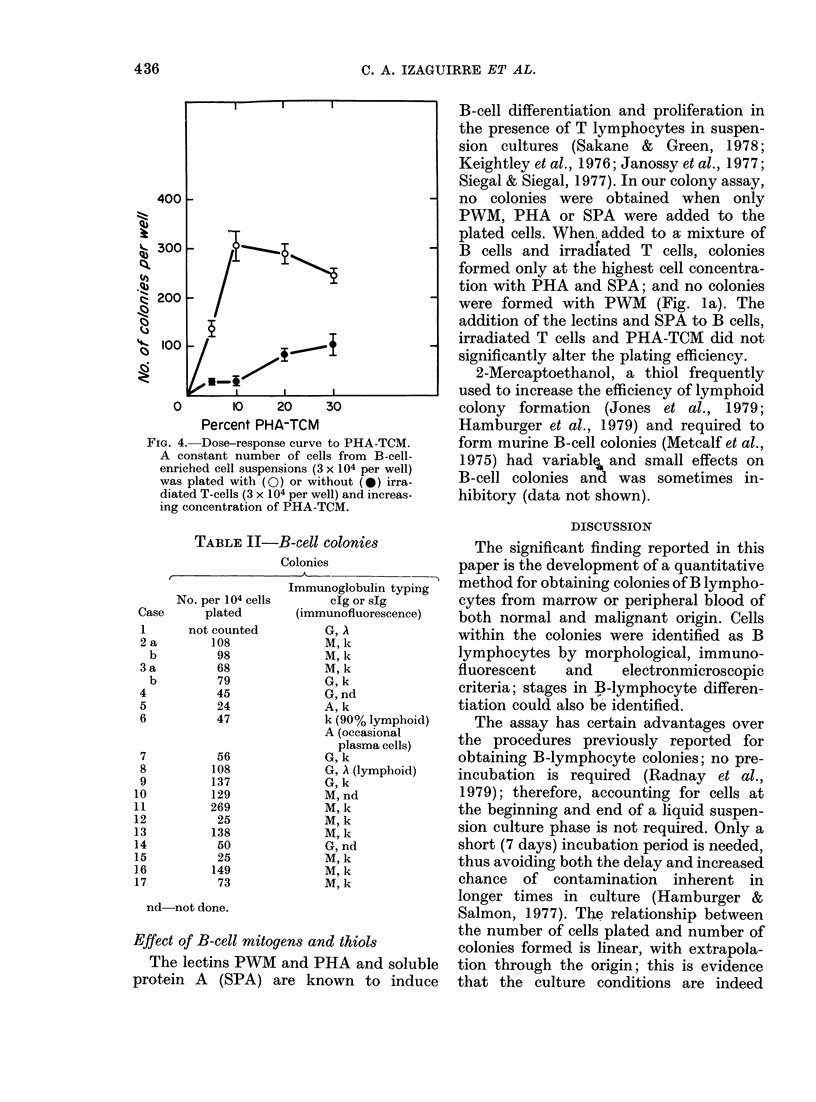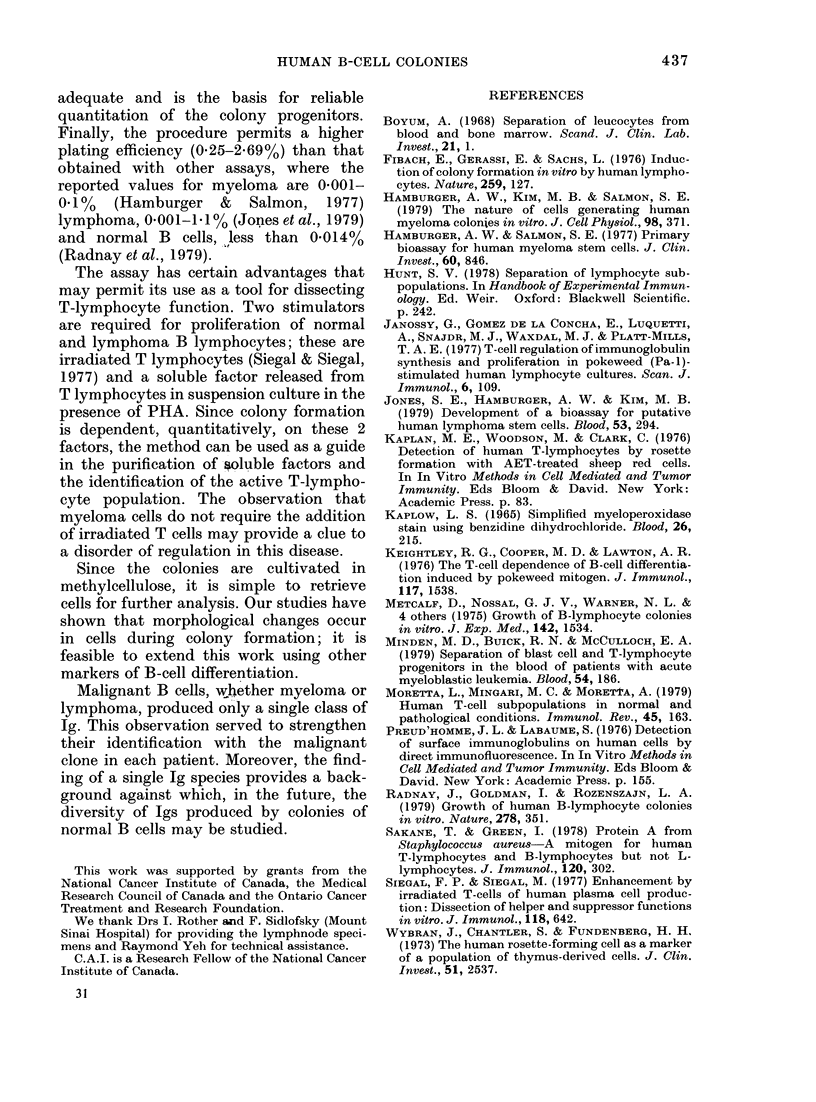# Colony formation by normal and malignant human B-lymphocytes.

**DOI:** 10.1038/bjc.1980.255

**Published:** 1980-09

**Authors:** C. A. Izaguirre, M. D. Minden, A. F. Howatson, E. A. McCulloch

## Abstract

**Images:**


Br. J. Cancer (1980) 42, 430

### COLONY FORMATION BY NORMAL AND MALIGNANT HUMAN B-LYMPHOCYTES

C. A. IZAGUIRRE, M. D. MINDEN, A. F. HOWATSON AND E. A. McCULLOCH

*From the School of Graduate Studies, University of Toronto, and The Ontario Cancer Institute, Toronto, Ontario, Canada*

Received 11 March 1980 Accepted 16 May 1980

**Summary.—A method is described that permits colony formation in culture by B lymphocytes from normal blood and from blood, marrow or lymph nodes of patients with myeloma or lymphoma. The method depends on: (1) exhaustively depleting cell suspensions of T lymphocytes, (2) a medium conditioned by T lymphocytes in the presence of phytohaemagglutinin (PHA-TCM), and (3) irradiated autologous or homologous T lymphocytes. Under these conditions the assay is linear. Cellular development of B lymphocytes can be followed; differentiation to plasma cells is seen in cultures of cells from normal individuals and myeloma patients, but not lymphoma patients. Malignant B lymphocytes in culture produced immunoglobulin of the class identified in the patient's blood, or in freshly obtained cells. We conclude that the assay is suitable for studying the growth, differentiation and regulation of normal and malignant B lymphocytes in culture.**

A NUMBER of methods have been described for obtaining colonies of normal or malignant B lymphocytes in culture. All of these have disadvantages; for example, the techniques of Fibach *et al.* (1976) for growing lymphoid colonies containing T cells and B cells from human peripheral blood, and that of Radnay *et al.* (1979) for growing normal and chronic lymphocytic leukaemia B cells require preincubation with pokeweed mitogen or phytohaemagglutinin (PHA) followed by plating in agar. Population changes during the preincubation phase made it difficult to obtain reliable quantitative data with those methods. Hamburger & Salmon (1977) have described a method for growing immunoglobulin-producing myeloma cells (malignant B cells). Their method requires a stimulator derived from cultures of adherent spleen cells from mice previously treated with mineral oil, and 18–21 days of incubation. Growth of myeloma cells was not always obtained with this technique, and the plating efficiency was routinely $<0 \cdot 1\%$.

In this paper we describe a method that permits colony formation by either normal or malignant B lymphocytes by the same technique and without pre-incubation; the method also mirrors, at least to some extent, the known cellular interactions involved in the regulation of immune responses (Moretta *et al.*, 1979). The method depends on the use, as stimulators, of medium conditioned by populations of semi-purified T cells and as well as irradiated T cells. It permits the development of colonies in 7 days with a plating efficiency that usually exceeds 1%.

### MATERIALS AND METHODS

### *Clinical material*

Blood, marrow or lymph nodes were obtained from patients with myeloma, B-cell acute lymphoblastic leukaemia (B-ALL), hairy-cell leukaemia and non-Hodgkin's

Repring requests: Dr E. A. McCulloch, Ontario Cancer Institute, 500 Sherbourne Street, Toronto, Ontario M4X 1K9 Canada.

TABLE I.—*Clinical material*

| Pt. | Diagnosis and clinical status | | Tissue studied | M-protein | Immunoglobulin typing, sIg or cIg (immuno-fluorescence) |
|---|---|---|---|---|---|
| 1 | Multiple myeloma | relapse | marrow | G, λ | G, λ |
| 2 a | Multiple myeloma | relapse | marrow | none | M, k |
| b | Multiple myeloma | relapse | marrow | none | M, k |
| 3 a | Multiple myeloma | new case | marrow | G, k | G, k |
| b | Multiple myeloma | relapse | marrow | G, k | G, k |
| 4 | Multiple myeloma | new case | marrow | G, λ | G, λ |
| 5 | Multiple myeloma | new case | marrow | A, k | A, k |
| 6 | Multiple myeloma | new case | marrow | 2 peaks A k | 80% k (lymphoid) 10% A (plasma cells) |
| 7 | Multiple myeloma | relapse | marrow | G, k | G, k |
| 8 | Plasmacytoma | relapse | marrow | none | G, λ (lymphoid) |
| 9 | Multiple myeloma | relapse | marrow | G, k | G, k |
| 10 | B-cell ALL | new case | blood | — | M, nd |
| 11 | Hairy-cell leukaemia | new case | blood | — | M, k |
| 12 | American Burkitt's lymphoma | new case | blood | — | M, k |
| 13 | NHL* | leukaemic transformation | marrow | — | M, k |
| 14 | NHL | leukaemic transformation | marrow | — | G, nd |
| 15 | NHL | leukaemic transformation | marrow | — | M, k |
| 16 | Diffuse histiocytic lymphoma | new case | lymph node | — | M, k |
| 17 | Mixed cellularity lymphoma | new case | lymph node | — | M, k |

* NHL—non-Hodgkin's lymphoma.

nd—not done.

lymphoma. The number of patients in each diagnostic category, and the source of cells, is shown in Table I. As controls, venous blood was obtained from laboratory workers.

In each instance, the cells were shown to belong to the B-cell lineage by immunofluorescence using a panel of specific antibodies against $\gamma$, $\mu$, $\alpha$, $\kappa$ and $\lambda$ chains; standard methods were used to demonstrate cytoplasmic (cIg) or surface immunoglobulin (sIg) (Preud'homme & Labaume, 1976). These data are also included in Table I. For the patients with myeloma, the immunoglobulin class found in association with the cells correlated with the M protein in sera, except for 2 cases (Table I) where M protein was not present.

Blood and marrow were collected in heparinized syringes; lymph nodes were obtained by biopsy and placed immediately in α-MEM (Gibco). Cell suspensions were prepared from the latter by mincing the tissue and then teasing small fragments apart with needles. Mononuclear cells were separated from suspensions of blood, marrow or lymph-node cells by centrifugation through Ficoll–Hypaque at a density of 1·077 (Boyum, 1968). Cells from patients with myeloma were cultured at this stage in the process without further cell separation. Cells from normal volunteers were depleted of adherent cells by incubation in Petri dishes in α-MEM, 20% foetal calf serum (FCS, Flow Labs) for 1 h at 37°C. Then, T lymphocytes were removed from the populations as described previously (Minden *et al.*, 1979) by using sheep erythrocytes (SRBC) to form rosettes (Wybran *et al.*, 1973) and removing these rosettes by centrifugation in Ficoll–Hypaque. In some instances, SRBCs treated with 2–5 aminoethylisothiouronium (AET, Sigma) were used (Kaplan *et al.*, 1976). The results with these separation procedures were equivalent and data obtained by this modification will not be presented separately. After T-cell depletion the cell suspensions contained B lymphocytes, non-T, non-B lymphocytes, monocytes and $<2\%$ T-cells. The cell suspensions so constituted were washed in α-MEM and 10% FCS (growth medium) and resuspended.

To obtain T-cell populations, cells that had formed SRBC rosettes were treated with $NH_4Cl$-tris buffer (Hunt, 1978) washed and resuspended in growth medium.

*Culture procedures*

Conditioned media were prepared by incubating suspensions of T cells, prepared as described above, with 1% PHA in growth medium at 37°C, for 2 or 3 days in a moist atmosphere with 5% $CO_2$. Supernatants of such cultures were collected, filter sterilized, and stored at 4°C. Such preparations were termed PHA-T-cell conditioned medium or PHA-TCM. Feeder cells consisted of purified autologous or homologous T lymphocytes irradiated with 20 Gy, using a caesium irradiator. In some experiments, T lymphocytes were treated with mitomycin C (50 $\mu$g/ml, Sigma) for 30 min at 37°C washed ×3 and used as feeder cells.

Cells from the B-cell enriched fraction were prepared at concentrations from $5 \times 10^4$ to $4 \times 10^5$ cells/ml in the presence of $3 \times 10^5$/ml irradiated T cells, 20% PHA-TCM, 0·8% methylcellulose and growth medium. After mixing the suspensions with a vortex mixer, 0·1ml aliquots were dispersed into wells of 96-well microtitre plates (Titertek, Limbro) flat bottomed, 0·6 mm in diameter, at least 4 replicates were plated for each cell concentration. The plates were covered and the covers secured tightly with masking tape. The cultures were then incubated at 37°C for 7 days in a moist atmosphere containing 5% $CO_2$. Where cytoplasmic immunofluorescence measurements were required, phenol red was omitted from α-MEM.

*Assessment of cultures*

Colonies containing more than 20 cells were counted on Day 5 and removed from culture for characterization subsequently up to Day 7. Individual colonies were picked using finely drawn Pasteur pipettes containing a small volume of phosphate buffered saline (PBS) diluted 1:1 with distilled water. Cells from single colonies were placed on a coverslip and centrifuged with a table-top centrifuge for 2 min, or a number of colonies were pooled. Cells from single colonies were assessed for morphology using Wright–Giemsa and for myeloperoxidase using the method of Kaplow (1965). Cells from single colonies were also tested for rosette formation with SRBC (Minden *et al.*, 1979). Cells pooled from 50–100 colonies were washed in PBS with 2% BSA and 0·2% sodium azide and then stained for cytoplasmic (cIg) or surface (sIg) immunoglobulin using standard techniques (Preud'homme & Labaume, 1976). For this purpose, labelled antibodies were purchased: Fluorescein-goat F(ab')$_2$ anti-human IgG F(ab')$_2$ fragment (polyvalent), fluorescein goat IgG anti-human G, A chain and M chain respectively (Cappel Lab.); fluorescein-donkey IgG anti-human kappa chain and fluorescein-goat IgG anti-human lambda chain (Meloy Lab.). Specificity was tested using a panel of multiple myeloma cells or leukaemic B cells that showed clonal staining.

*Mitogens.*—Phytohaemagglutinin (PHA) (Wellcome H-15) was used at 0·5 or 1%. Pokeweed mitogen (PWM) (Gibco) was used at 1% dilution of stock, soluble protein A (SPA) from *Staph. aureus* was purchased from Pharmacia and used at 50 $\mu$g/ml. 2-Mercaptoethanol (Sigma) $5 \times 10^{-5}$M was added to cultures in some experiments.

*Electron microscopy.*—Colonies were picked sequentially between 3 to 8 days of culture, centrifuged through horse serum, diluted 50% with α-MEM, fixed with 3·5% glutaraldehyde in cacodylate buffer and processed by standard techniques for thin-section ultrastructural studies.

### RESULTS

*Colony formation*

When suspensions of B cells, prepared as described in Materials and Methods, are plated under optimal conditions in wells, 2- and 4-cell clusters were seen after 24 h. By the 3rd day there were discrete colonies: these enlarged until Day 7 and became confluent and/or disintegrated. The colonies were of variable size, usually tight with some loosely attached cells at the edge of the colony. They contained from 20–500 cells (Fig. 1a). Cells within the colonies were characterized during the process of growth. By Day 3 they looked like large lymphoblasts as seen in the Wright–Giemsa preparations, but were negative for cIg. From Day 4 to Day 6 cells within colonies were heterogeneous; some continued to look like lymphoblasts while others had the characteristics of immature plasma cells by electron microscopy (Fig. 2) and Wright-Giemsa (Fig. 1b). Cytoplasmic Ig was usually first detected on Day 5 and staining for cIg reached maximum intensity by Day 7; at

FIG. 1.—Characterization of normal B-cell colonies (×100). (a) B-cell colonies Day 7. (b) Wright-Giemsa stained cells. (c) $sIg^+$ cells. (d) $cIg^+$ cells.

this time most, but not all, cells were $sIg^+$ (Fig. 1c) and typical plasma cells were seen (Figs 1d and 2b).

This sequence of events was observed for colony formation by cells from both normals and patients with B-cell malignancies; however, cells from marrow, blood or lymph nodes of lymphoma or B-cell leukaemia patients formed colonies that contained cells which remained lymphoid with scant cIg; plasma cells were seen in multiple-myeloma colonies. No peroxidase-positive cells were detected in single or pooled colony staining.

*Quantitation*

The assay could be used quantitatively, since a linear relationship was observed between cell number plated and colony formation, which was critically dependent upon culture conditions. At high cell densities no colonies were seen in cultures containing methyl-cellulose and growth medium only. The addition of PHA-TCM induced colony formation. But colony formation at the low cell densities required for the quantitative assay was achieved only in cultures with both PHA-TCM and irradiated autologous or homologous T-cell feeders. Fig. 3 shows the effect of adding irradiated feeders to cultures of normal B cell (3a), B-cell leukaemia cells (3b), cells from lymphoma lymph nodes (3c) and myeloma cells (3d). Only in multiple myeloma was a requirement for added feeders not found. Mitomycin C-treated T cells were as effective as irradiated T cells in normal B-cell colony formation.

Fig. 4 demonstrates the importance of PHA-TCM; in this experiment cells were plated with increasing concentrations of PHA-TCM in the presence or absence of irradiated T-cell feeders. It is apparent that optimal plating efficiency was obtained only when both stimulators were present and at concentrations of PHA-TCM $>10\%$. Not only was the number of colonies increased with increasing stimu-

FIG. 2.—Ultrastructure of B-cell colonies. (a) an immature cell with rough endoplasmic reticulum (Day 4). (b) a characteristic plasma cell (Day 7).

FIG. 3.—An increasing number of B cells was plated with (○) or without (●) a constant number of irradiated T-cells ($3 \times 10^4$ per well) and 20% PHA-TCM. (a) includes substitution of PHA-TCM by SPA (△), PHA (□) and PWM (■).

lator concentration, their size was greater. A concentration of 20% PHA-TCM was selected as optimum.

*Immunoglobulin distributions in malignant B-cell colonies*

Immunoglobulin classes were characterized in the cells of origin of colonies and in the cells from colonies. The data are given in Table II. It is apparent that the same Ig class was found both in freshly obtained cells and in cells from colonies. Further evidence for the origin of malignant cells was obtained in Case 1. Metaphases from fresh marrow and from colonies showed 48 chromosomes.

FIG. 4.—Dose-response curve to PHA-TCM. A constant number of cells from B-cell-enriched cell suspensions ($3 \times 10^4$ per well) was plated with (○) or without (●) irradiated T-cells ($3 \times 10^4$ per well) and increasing concentration of PHA-TCM.

TABLE II—*B-cell colonies*

| Case | Colonies: No. per $10^4$ cells plated | Colonies: Immunoglobulin typing cIg or sIg (immunofluorescence) |
|---|---|---|
| 1 | not counted | G, λ |
| 2 a | 108 | M, k |
| b | 98 | M, k |
| 3 a | 68 | M, k |
| b | 79 | G, k |
| 4 | 45 | G, nd |
| 5 | 24 | A, k |
| 6 | 47 | k (90% lymphoid) A (occasional plasma cells) |
| 7 | 56 | G, k |
| 8 | 108 | G, λ (lymphoid) |
| 9 | 137 | G, k |
| 10 | 129 | M, nd |
| 11 | 269 | M, k |
| 12 | 25 | M, k |
| 13 | 138 | M, k |
| 14 | 50 | G, nd |
| 15 | 25 | M, k |
| 16 | 149 | M, k |
| 17 | 73 | M, k |

nd—not done.

### *Effect of B-cell mitogens and thiols*

The lectins PWM and PHA and soluble protein A (SPA) are known to induce B-cell differentiation and proliferation in the presence of T lymphocytes in suspension cultures (Sakane & Green, 1978; Keightley *et al.*, 1976; Janossy *et al.*, 1977; Siegal & Siegal, 1977). In our colony assay, no colonies were obtained when only PWM, PHA or SPA were added to the plated cells. When added to a mixture of B cells and irradiated T cells, colonies formed only at the highest cell concentration with PHA and SPA; and no colonies were formed with PWM (Fig. 1a). The addition of the lectins and SPA to B cells, irradiated T cells and PHA-TCM did not significantly alter the plating efficiency.

2-Mercaptoethanol, a thiol frequently used to increase the efficiency of lymphoid colony formation (Jones *et al.*, 1979; Hamburger *et al.*, 1979) and required to form murine B-cell colonies (Metcalf *et al.*, 1975) had variable and small effects on B-cell colonies and was sometimes inhibitory (data not shown).

### DISCUSSION

The significant finding reported in this paper is the development of a quantitative method for obtaining colonies of B lymphocytes from marrow or peripheral blood of both normal and malignant origin. Cells within the colonies were identified as B lymphocytes by morphological, immunofluorescent and electronmicroscopic criteria; stages in B-lymphocyte differentiation could also be identified.

The assay has certain advantages over the procedures previously reported for obtaining B-lymphocyte colonies; no preincubation is required (Radnay *et al.*, 1979); therefore, accounting for cells at the beginning and end of a liquid suspension culture phase is not required. Only a short (7 days) incubation period is needed, thus avoiding both the delay and increased chance of contamination inherent in longer times in culture (Hamburger & Salmon, 1977). The relationship between the number of cells plated and number of colonies formed is linear, with extrapolation through the origin; this is evidence that the culture conditions are indeed adequate and is the basis for reliable quantitation of the colony progenitors. Finally, the procedure permits a higher plating efficiency (0·25–2·69%) than that obtained with other assays, where the reported values for myeloma are 0·001–0·1% (Hamburger & Salmon, 1977) lymphoma, 0·001–1·1% (Jones *et al.*, 1979) and normal B cells, less than 0·014% (Radnay *et al.*, 1979).

The assay has certain advantages that may permit its use as a tool for dissecting T-lymphocyte function. Two stimulators are required for proliferation of normal and lymphoma B lymphocytes; these are irradiated T lymphocytes (Siegal & Siegal, 1977) and a soluble factor released from T lymphocytes in suspension culture in the presence of PHA. Since colony formation is dependent, quantitatively, on these 2 factors, the method can be used as a guide in the purification of soluble factors and the identification of the active T-lymphocyte population. The observation that myeloma cells do not require the addition of irradiated T cells may provide a clue to a disorder of regulation in this disease.

Since the colonies are cultivated in methylcellulose, it is simple to retrieve cells for further analysis. Our studies have shown that morphological changes occur in cells during colony formation; it is feasible to extend this work using other markers of B-cell differentiation.

Malignant B cells, whether myeloma or lymphoma, produced only a single class of Ig. This observation served to strengthen their identification with the malignant clone in each patient. Moreover, the finding of a single Ig species provides a background against which, in the future, the diversity of Igs produced by colonies of normal B cells may be studied.

This work was supported by grants from the National Cancer Institute of Canada, the Medical Research Council of Canada and the Ontario Cancer Treatment and Research Foundation.

We thank Drs I. Rother and F. Sidlofsky (Mount Sinai Hospital) for providing the lymphnode specimens and Raymond Yeh for technical assistance.

C.A.I. is a Research Fellow of the National Cancer Institute of Canada.

REFERENCES

Boyum, A. (1968) Separation of leucocytes from blood and bone marrow. *Scand. J. Clin. Lab. Invest.*, **21**, 1.

Fibach, E., Gerassi, E. & Sachs, L. (1976) Induction of colony formation *in vitro* by human lymphocytes. *Nature*, **259**, 127.

Hamburger, A. W., Kim, M. B. & Salmon, S. E. (1979) The nature of cells generating human myeloma colonies *in vitro*. *J. Cell Physiol.*, **98**, 371.

Hamburger, A. W. & Salmon, S. E. (1977) Primary bioassay for human myeloma stem cells. *J. Clin. Invest.*, **60**, 846.

Hunt, S. V. (1978) Separation of lymphocyte subpopulations. In *Handbook of Experimental Immunology*. Ed. Weir. Oxford: Blackwell Scientific. p. 242.

Janossy, G., Gomez de la Concha, E., Luquetti, A., Snajdr, M. J., Waxdal, M. J. & Platt-Mills, T. A. E. (1977) T-cell regulation of immunoglobulin synthesis and proliferation in pokeweed (Pa-1)-stimulated human lymphocyte cultures. *Scan. J. Immunol.*, **6**, 109.

Jones, S. E., Hamburger, A. W. & Kim, M. B. (1979) Development of a bioassay for putative human lymphoma stem cells. *Blood*, **53**, 294.

Kaplan, M. E., Woodson, M. & Clark, C. (1976) Detection of human T-lymphocytes by rosette formation with AET-treated sheep red cells. In In Vitro *Methods in Cell Mediated and Tumor Immunity*. Eds Bloom & David. New York: Academic Press. p. 83.

Kaplow, L. S. (1965) Simplified myeloperoxidase stain using benzidine dihydrochloride. *Blood*, **26**, 215.

Keightley, R. G., Cooper, M. D. & Lawton, A. R. (1976) The T-cell dependence of B-cell differentiation induced by pokeweed mitogen. *J. Immunol.*, **117**, 1538.

Metcalf, D., Nossal, G. J. V., Warner, N. L. & 4 others (1975) Growth of B-lymphocyte colonies *in vitro*. *J. Exp. Med.*, **142**, 1534.

Minden, M. D., Buick, R. N. & McCulloch, E. A. (1979) Separation of blast cell and T-lymphocyte progenitors in the blood of patients with acute myeloblastic leukemia. *Blood*, **54**, 186.

Moretta, L., Mingari, M. C. & Moretta, A. (1979) Human T-cell subpopulations in normal and pathological conditions. *Immunol. Rev.*, **45**, 163.

Preud'homme, J. L. & Labaume, S. (1976) Detection of surface immunoglobulins on human cells by direct immunofluorescence. In In Vitro *Methods in Cell Mediated and Tumor Immunity*. Eds Bloom & David. New York: Academic Press. p. 155.

Radnay, J., Goldman, I. & Rozenszajn, L. A. (1979) Growth of human B-lymphocyte colonies *in vitro*. *Nature*, **278**, 351.

Sakane, T. & Green, I. (1978) Protein A from *Staphylococcus aureus*—A mitogen for human T-lymphocytes and B-lymphocytes but not L-lymphocytes. *J. Immunol.*, **120**, 302.

Siegal, F. P. & Siegal, M. (1977) Enhancement by irradiated T-cells of human plasma cell production: Dissection of helper and suppressor functions *in vitro*. *J. Immunol.*, **118**, 642.

Wybran, J., Chantler, S. & Fundenberg, H. H. (1973) The human rosette-forming cell as a marker of a population of thymus-derived cells. *J. Clin. Invest.*, **51**, 2537.